# Dysregulation of Monounsaturated Fatty Acids is Related to α‐Synuclein in Multiple System Atrophy

**DOI:** 10.1002/mds.30248

**Published:** 2025-05-23

**Authors:** Finula I. Isik, YuHong Fu, Russell Pickford, Qi Cheng, Yue Yang, Simon J.G. Lewis, Nicolas Dzamko, Glenda M. Halliday, Woojin Scott Kim

**Affiliations:** ^1^ Brain and Mind Centre The University of Sydney Sydney NSW Australia; ^2^ School of Medical Sciences The University of Sydney Sydney NSW Australia; ^3^ Bioanalytical Mass Spectrometry Facility University of New South Wales Sydney NSW Australia; ^4^ Department of Neurology Jiangwan Hospital of Hongkou District Shanghai China; ^5^ Macquarie Medical School, Faculty of Medicine, Health and Human Sciences Macquarie University Sydney NSW Australia

**Keywords:** multiple system atrophy, α‐synuclein, monounsaturated fatty acids, stearoyl‐CoA desaturase, phospholipid

## Abstract

**Background:**

Multiple system atrophy (MSA) is a neurodegenerative disease pathologically characterized by the presence of glial cytoplasmic inclusions (GCI) composed of α‐synuclein aggregates. In Parkinson's disease, increases in monounsaturated fatty acids (MUFA) in phospholipid membranes promote α‐synuclein binding, aggregation, and toxicity, and the inhibition of stearoyl‐CoA desaturase (SCD), the enzyme responsible for synthesizing MUFA, alleviates α‐synuclein toxicity. However, little is known about phospholipid MUFA or SCD in the context of MSA pathology.

**Objectives:**

To determine whether phospholipid MUFA and SCD levels are altered in MSA brain and related to α‐synuclein pathology.

**Methods:**

Phospholipid MUFA levels in the disease‐affected motor cortex white matter (MWM) and disease‐unaffected superior occipital cortex (SOC) of postmortem MSA and control brain were analyzed using liquid chromatography–mass spectrometry. Brain GCI, α‐synuclein, and SCD were analyzed using immunofluorescence, Western blotting, and quantitative polymerase chain reaction (qPCR). Serum SCD was analyzed using ELISA.

**Results:**

MUFA in phosphatidic acid, phosphatidylcholine, and phosphatidylethanolamine were elevated in MSA MWM compared with control MWM by 3.9%, 8.8%, and 9.5%, respectively, whereas none were altered in SOC. MUFA were strongly associated with α‐synuclein only in MWM. SCD mRNA and protein expression were decreased only in MSA MWM compared with control MWM.

**Conclusions:**

These findings suggest a prevalence of MUFA dysregulation in specific regions of MSA brain, resulting in MUFA levels remaining high despite decreases in SCD expression. Our study has provided new insights into an unrecognized pathway in MSA and opened a new area of research for better understanding MSA pathogenesis. © 2025 The Author(s). *Movement Disorders* published by Wiley Periodicals LLC on behalf of International Parkinson and Movement Disorder Society.

## 1 Introduction

Multiple system atrophy (MSA) is a rapidly progressing neurodegenerative disease affecting nigrostriatal, olivopontocerebellar, and autonomic brainstem structures. MSA is estimated to affect 1.6–3 in 100,000 age‐adjusted individuals.^1^, ^2^ Along with the α‐synuclein Lewy body diseases Parkinson's disease (PD) and dementia with Lewy bodies (DLB), it is classified as a synucleinopathy. However, unlike PD or DLB, MSA is neuropathologically defined by the presence of α‐synuclein‐positive glial cytoplasmic inclusions (GCI) in oligodendrocytes^3^, ^4^ and is more aggressive in nature.^5^ Oligodendroglial myelin lipids in particular are dysregulated in disease‐affected regions of the brain,^6^ with increases in unsaturated lipids (those containing one or more carbon–carbon double bonds) prone to peroxidation by reactive oxygen species resulting in the formation of toxic aldehydes.^7^

Emerging data indicate that increases in monounsaturated fatty acids (MUFA; contain one carbon–carbon double bond) in phospholipids promote α‐synuclein binding to membranes, misfolding, and toxicity.^8^, ^9^, ^10^, ^11^ MUFA are synthesized by the enzyme stearoyl‐CoA desaturase (SCD), which introduces a cis double bond at n‐9 in saturated fatty acids, generating, predominantly, palmitoleate (C16:1) and oleate (C18:1) that incorporate into phospholipids, triglycerides, and cholesteryl esters.^12^ Inhibition of SCD reduces the desaturation index of fatty acids (the ratio of monounsaturated to saturated fatty acids) and as a result alleviates α‐synuclein binding, aggregation, and toxicity in vitro^8^, ^13^, ^14^, ^15^, ^16^, ^17^, ^18^, ^19^ and in vivo.^9^, ^10^, ^12^ In fact, the SCD inhibitor YTX‐7739, which decreases the fatty acid desaturation index in rodents and monkeys,^20^ has now advanced to a phase 1 clinical trial for the treatment of PD (https://www.onderzoekmetmensen.nl/en/trial/20317).

Despite the progress in understanding the detrimental effects of MUFA and the possible clinical benefits of inhibiting SCD activity in PD, very little is known about MUFA and SCD in the context of MSA pathology. MUFA are prominent components of lipids, such as phospholipids, sphingomyelin, and sulfatides, which are important in myelin function.^21^ Consistent with this, SCD is highly expressed in mature oligodendrocytes, indicating the importance of MUFA in myelin formation.^21^ In another neurological disease that affects myelin, multiple sclerosis (MS), SCD, along with other myelin‐associated proteins, are decreased in brain lesions.^22^ We hypothesize that MUFA and SCD are altered in MSA brain contributing to MSA pathogenesis. We therefore assessed MUFA, SCD, and α‐synuclein in different regions of postmortem MSA and control brain to investigate how MUFA could be related to α‐synuclein pathology.

## 2 Methods

### 2.1 Human Brain Tissues

Frozen brain tissues were obtained from the Sydney Brain Bank and NSW Brain Tissue Resource Centre. Ethical approval was acquired from the human research ethics committee of the University of Sydney (Approval Number: 2019/589). Samples included the disease‐affected motor cortex white matter (MWM) and disease‐unaffected superior occipital cortex (SOC) from MSA cases (N = 11, male = 7, female = 4) and controls (N = 13, male = 6, female = 7) without neurological, psychiatric, or neuropathological diagnoses. The mean age at death ± standard error of the mean (SEM), median age, and variance for MSA was 71.1 ± 2.3, 71, and 56.3 years, and for controls 90.7 ± 2.2, 89, and 62.7 years. The F‐test value was 1.114 at *P* = 0.8761.

### 2.2 Human Serum

Blood serum samples were obtained from participants diagnosed with MSA (N = 10, male = 6, female = 4) or PD, a positive control for synucleinopathy, (N = 13, male = 8, female = 5). Progressive supranuclear palsy (PSP) (N = 16, male = 13, female = 3) cases were included as a negative control for synucleinopathy. Participants were recruited with informed consent from the Parkinson's disease research clinic at the Brain and Mind Centre, The University of Sydney. The cases were diagnosed according to clinically established criteria,^23^ which included motor assessments (Movement Disorder Society‐ Unified Parkinson's Disease Rating Scale‐Part III [MDS‐UPDRS‐III] scores and Hoehn and Yahr [H&Y] stage). Age‐ and sex‐matched controls (N = 12, male = 8, female = 4) had no neurological or psychiatric disorders. The mean and median ages for MSA were 64.2 and 64.3 years, PD 68.2 and 66.3 years, PSP 69.2 and 68.0 years, and controls 65.5 and 68.0 years. Blood samples were obtained following written informed consent from the participants. Serum from each participant was collected in 8.5 mL Serum Separator Tube II Advanced tubes (BD Biosciences). Serum samples were incubated at room temperature for 30 min to allow blood to clot and were then gently inverted five times prior to centrifugation at 1500 × *g* for 15 min at 21°C (acceleration at 9 and deceleration at 5). Serum was then aliquoted (500 μL aliqouts) and stored at −80°C until required for experiments. The study was approved by the University of Sydney Human Research Ethics Committee (Approval Number: 2017/826).

### 2.3 Chemicals and Materials

Lipids were extracted using chloroform or methyl‐tert‐butyl ether (MTBE), methanol (MeOH), and isopropanol (Sigma Aldrich, St. Louis, MO, USA) and ultrapure water (Millipore). All solvents used were high‐performance liquid chromatography grade or higher. Glass pipettes and tubes were used wherever possible and the use of plasticware was minimized during lipid extraction to avoid contamination of samples. Glass tubes and glass transfer pipettes were purchased from Sigma and VWR. Lipid internal standards (ISTD) were purchased from Avanti Polar Lipids Inc. (Alabaster, AL, USA). These include phosphatidylcholine (19:0), sphingomyelin (12:0), phosphatidylethanolamine (17:0), phosphatidylglycerol (17:0), phosphatidylserine (17:0), phosphatidic acid (17:0), ceramide (d18:1, 12:0), diglyceride (1,3 18:0 d5), cholesteryl ester (19:0), monoglyceride (17:0), triglyceride mix d5 (Avanti Code LM‐6000), diglyceride mix d5 (Avanti Code LM‐6001), phosphatidylinositol (17:0 14:1), C12 GluCer, C12 sulfatide, C17 ceramide, C17 sphingosine, C17 S1P, C12 C1P, D3 C20 fatty acid, and C12 LacCer. Lipid internal standards were prepared as a mixture at 10 pmol/μL in MTBE and MeOH (MTBE:MeOH 1:1 v/v).

### 2.4 Lipid Extraction

Brain tissue lipid extraction was based on the Matyash method.^24^ Briefly, 20 mg of fresh‐frozen brain tissues were homogenized together with 10 μL of the ISTD mix in MeOH containing 0.01% butylated hydroxytoluene (300 μL) using a Qiagen TissueLyser (3 × 30 s, 30 Hz cycles). The homogenates were transferred to glass tubes, as well as the MeOH (600 μL) wash of the beads. MTBE (3.5 mL) was added and the mixture vortexed and incubated for 1 hr at room temperature. Water (875 μL) was added and the mixture vortexed and centrifuged at 1000 g for 10 min. The upper phase was transferred to a new glass tube using a glass Pasteur pipette. The lower phase was re‐extracted using MTBE/MeOH/water (10:3:2.5). The preparation was dried under nitrogen gas. Dried lipid samples were reconstituted in 100 μL of chloroform/ MeOH (1:1) and stored at −80°C in glass liquid chromatography–mass spectrometry (LC–MS) vials.

### 2.5 LC–MS

Lipid extracts (10 μL) were analyzed using a Q‐Exactive HF Mass Spectrometer coupled to a U3000 UPLC system (ThermoFisher Scientific). Chromatography was performed at 60°C on a Waters CSH C18 UHPLC column 2.1 × 100 mm, 1.8 μM with VanGuard guard column. Solvent A was 6:4 acetonitrile:water and Solvent B was 1:9 acetonitrile:isopropanol, both with 10 mM ammonium formate and 0.1% formic acid. Lipids were chromatographed according to the method of Castro‐Perez et al.^25^ Briefly, a 30‐min gradient running from 30% to 100% of solvent B was performed, eluting lipids in order of hydrophobicity. Column eluate was directed into the electrospray ionization source of the mass spectrometer where a heated‐electrospray ionization probe was employed. Source parameters were broadly optimized on a range of lipid standards prior to the analysis. The mass spectrometer was run in data‐dependent acquisition mode. A survey scan over the mass range 200–1500 at resolution 120 K was followed by 20 data‐dependent MS/MS scans on the most intense ions in the survey at 15 K resolution. Dynamic exclusion was used to improve the number of ions targeted. Cycle time was approximately 1 s. Samples were run in both positive and negative polarities. The samples were run in a random order (generated using Microsoft Excel). This was important to avoid batch effects/changing instrument performance effects. Data were analyzed using LipidSearch software 4.2.29. Data were searched against the standard Lipidsearch database with all common mammalian lipid classes included. The search results were then grouped according to sample type and aligned for differential analysis. Aligned data (containing lipid identity, peak area, retention time, etc.) were exported to Excel software. Abundance of lipids was obtained from peak areas for each lipid species. They were normalized between samples, to correct for batch effects from the sample preparation and the LC–MS analysis, using the internal standards of the same lipid category. They were then normalized to the weight of the brain tissues used.

### 2.6 Immunofluorescence

Formalin‐fixed paraffin‐embedded tissues sections (4 μm) were de‐waxed with HistoChoice Clearing Agent (Sigma, H2779) and rehydrated with gradient concentrations of ethanol. Sections were treated with formic acid (70% for 20 min) and then underwent heat‐induced antigen retrieval in a programmable antigen retrieval cooker (Aptum Bio Retriever 2100, Aptum Biologics Ltd, Southampton, UK). After washing with phosphate‐buffered saline (PBS), the elimination of autofluorescence was performed using LED light (35,000 LUX), followed by 0.1% Sudan Black in 70% ethanol treatment. Sections were blocked with blocking buffer (PBS, 2% donkey serum, 1% bovine serum albumin) for 1 hr at room temperature. Primary antibody incubation against α‐synuclein phosphorylated on Ser129 (Abcam Ab51253, 1:100) and tubulin polymerization‐promoting protein (TPPP)/p25α (Thermo Fisher Scientific PA5‐19243, 1:100) was performed at appropriate dilutions for 24 hr, followed by Alexa Fluor 488 and 647 secondary antibodies, respectively, and 4′,6‐diamidino‐2‐phenylindole (DAPI; Sigma, cat#D9542, 1 mg/mL) for 2 hr at room temperature. Slides were treated with 10 mM Cu_2_SO_4_ to further quench autofluorescence before finally being cover‐slipped with mounting medium (DAKO, cat# S3023) and sealed with nail polish. Negative controls were performed for each staining batch that omitted primary or secondary antibodies.

### 2.7 Protein Extraction

Tris‐buffered saline (TBS) soluble and sodium dodecyl sulfate (SDS)‐soluble proteins were serially extracted from 50 mg of fresh‐frozen brain tissue as previously described.^26^ In brief, tissue samples were homogenized in 10 volumes of TBS homogenization buffer (20 mM Tris, 150 mM NaCl, pH 7.4, 5 mM EDTA, 0.02% sodium azide) containing protease inhibitor cocktail (Roche) using Qiagen Tissue Lyser (3 × 30 sec, 30 Hz cycles), followed by centrifugation at 100,000 × *g* for 1 hr at 4°C, with supernatant collected as the TBS‐soluble fraction containing cytosolic proteins. The pellet was resuspended in SDS solubilization buffer (TBS homogenization buffer containing 5% SDS) using 3 × 30 s, 30 Hz cycles with Qiagen Tissue Lyser, and centrifuged at 100,000 × *g* for 30 min at 25°C, with supernatant collected as the SDS‐soluble fraction containing membrane‐associated proteins. Protein concentration was measured using bicinchoninic acid assay (Pierce BCA Protein Assay Kit) following the manufacturer's instructions.

### 2.8 Western Blotting

Protein lysates (10 μg) were heated with sample buffer (3.2% SDS, 32% glycerol, 0.16% bromophenol blue, 100 mM Tris–HCl, pH 6.8, 8% 2‐mercaptoethanol). They were electrophoresed on Criterion stain free 4%–20% SDS‐polyacrylamide gel electrophoresis (SDS‐PAGE) gels (Bio‐Rad) and transferred onto nitrocellulose membranes at 90 volts for 90 min. The membranes were blocked with TBS containing 5% w/v non‐fat dry milk and probed with anti‐SCD antibody (Invitrogen, PA5‐19682, 1:1000) or anti‐α‐synuclein (19 kDa α‐synuclein, BD Biosciences, 610787, 1:2000) overnight at 4°C. They were then washed three times in TBS containing 0.1% Tween 20 and incubated with horseradish peroxidase‐conjugated secondary antibody for 2 hr at room temperature. Signals were detected using enhanced chemiluminescence and Gel Doc System (Bio‐Rad). The blots were stripped and probed for housekeeper protein β‐actin. The signal intensity was quantified using Image Lab (Bio‐Rad) and NIH ImageJ software (v1.45s).

### 2.9 RNA Isolation, Reverse Transcription, and Quantitative Polymerase Chain Reaction

RNA was extracted from tissues using TRIzol Reagent (Invitrogen) as per the manufacturer's protocol. All procedures were carried out using RNase‐free reagents and consumables. RNA (1 μg) was reverse‐transcribed into cDNA using Moloney‐murine leukemia virus (M‐MLV) reverse transcriptase, and random primers (Promega, Madison, WI, USA) in 20 μL reaction volume. Quantitative polymerase chain reaction (qPCR) assays were carried out using a Mastercycler ep realplex S (Eppendorf) and the fluorescent dye SYBR Green (Bio‐Rad), following the manufacturer's protocol. Briefly, each reaction (20 μl) contained 1 × mastermix, 5 pmol of primers and 1 μl of cDNA template. Amplification was carried out with 40 cycles of 94°C for 15 sec and 60°C for 1 min. Gene expression was normalized to the geometric mean of three housekeeper genes, GAPDH, β‐actin, and PPIA. A no‐template control was included for each PCR amplification assay. The level of expression for each gene was calculated using the comparative threshold cycle (Ct) value method using the formula 2^−ΔΔCt^ (where ΔΔCt = ΔCt sample − ΔCt reference).

### 2.10 SCD ELISA

Serum SCD was measured using an ELISA kit (OKEH07143; Aviva Systems Biology Corporation, San Diego, CA, USA) following the manufacturer's instructions. Serum samples were diluted 1:2 and the optical density (OD) read at 450 nm using CLARIOstar.

### 2.11 Statistical Analysis

The statistical analyses of postmortem brain tissues and serum were performed using IBM SPSS Statistics (version 26). All plots were generated using GraphPad Prism (Prism 10 for Windows, Version 10.4.1). Multivariate analysis (general linear model) covarying for age and sex was used to assess differences between proportion (%) of MUFA in phospholipids in MSA and controls, with the confidence interval set at 95% (95% CI) and significance determined at a *P‐*value (two‐tailed) < 0.05. There were no missing data, and statistics were based on all cases with valid data for all variables in the model. Corrections for multiple comparisons were not performed. Spearman correlation analyses were performed to determine associations between proportion (%) of MUFA in phospholipids and α‐synuclein protein expression (OD), with 95% CI and significance determined at a *P‐*value (two‐tailed) < 0.05. The results were presented as mean ± standard error of the mean (SEM).

## 3 Results

### 3.1 Phospholipid MUFA are Elevated in MSA Brain

**FIG 1:**
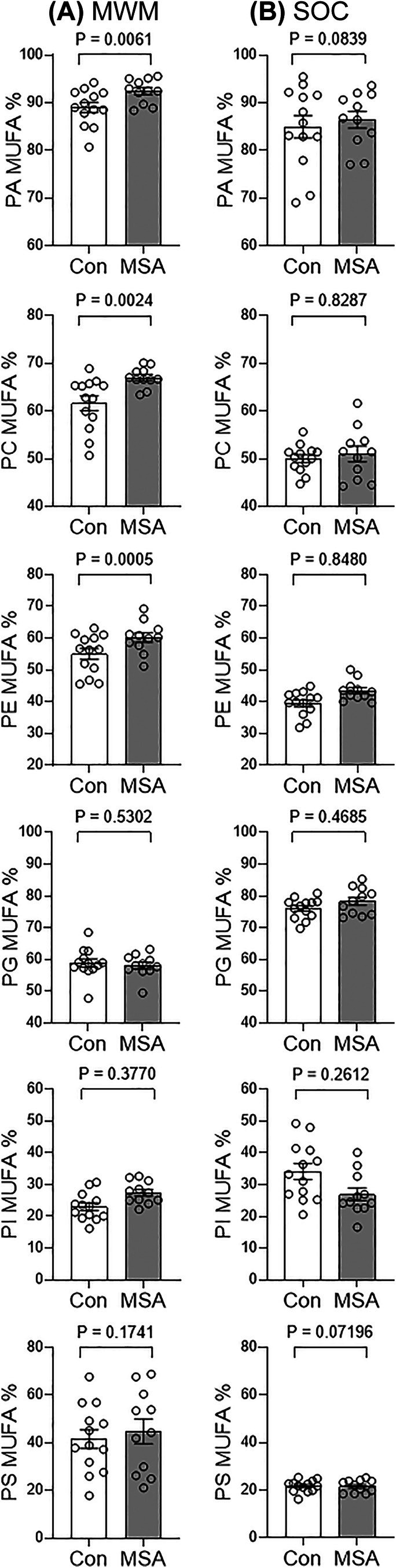
Assessment of monounsaturated fatty acids in phospholipids in multiple system atrophy (MSA) brain. (A) Relative proportion of monounsaturated fatty acids (MUFA) in phosphatidic acid (PA), phosphatidylcholine (PC), phosphatidylethanolamine (PE), phosphatidylglycerol (PG), phosphatidylinositol (PI), and phosphatidylserine (PS) (%) in MSA (N = 11) and controls (Con; N = 13) in the disease‐affected motor cortex white matter (MWM). PA Con = 89.02 ± 1.188, MSA = 92.45 ± 1.344, F = 9.388; PC Con = 61.63 ± 1.545, MSA = 67.04 ± 1.747, F = 12.053; PE Con = 55.00 ± 1.807, MSA = 60.21 ± 2.043, F = 16.898; PG Con = 58.89 ± 1.611, MSA = 58.09 ± 1.821, F = 0.408; PI Con = 23.05 ± 1.442, MSA = 27.43 ± 1.631, F = 0.816; PS Con = 41.61 ± 5.858, MSA = 44.79 ± 6.622, F = 1.986. (B) Relative proportion of MUFA in PA, PC, PE, PG, PI, and PS (%) in MSA (N = 11) and Con (N = 13) in the disease‐unaffected superior occipital cortex (SOC). PA Con = 84.97 ± 2.642, MSA = 86.43 ± 2.987, F = 3.310; PC Con = 50.06 ± 1.644, MSA = 51.02 ± 1.859, F = 0.048; PE Con = 39.44 ± 1.267, MSA = 43.51 ± 1.432, F = 0.038; PG Con = 76.06 ± 1.441, MSA = 78.36 ± 1.629, F = 0.546; PI Con = 34.11 ± 3.126, MSA = 26.99 ± 3.534, F = 1.337; PS Con = 21.74 ± 0.854, MSA = 21.70 ± 0.966, F = 3.610.

Very little is known about MUFA in relation to MSA pathology. We therefore undertook a comprehensive assessment of MUFA (ie, oleate and palmitoleate) in MSA brain tissues. Specifically, we measured MUFA incorporated in phospholipids, which are the major components of myelin and are pivotal to myelin function. We found that the proportion (%) of MUFA in phosphatidic acid (PA) (Con: 89.02, MSA: 92.45, *P* = 0.0061), phosphatidylcholine (PC) (Con: 61.63, MSA: 67.04, *P* = 0.0024), and phosphatidylethanolamine (PE) (Con: 55.00, MSA: 60.21, *P* = 0.0005) was significantly elevated in the disease‐affected MWM in MSA compared with controls (Fig. 1A). The proportion of MUFA in other phospholipids, namely phosphatidylglycerol (PG) (Con: 58.89, MSA: 58.09, *P* = 0.5302), phosphatidylinositol (PI) (Con: 23.05, MSA: 27.43, *P* = 0.3770), and phosphatidylserine (PS) (Con: 41.61, MSA: 44.79, *P* = 0.1741), was unaltered in MSA MWM compared with controls (Fig. 1A). We also measured phospholipid MUFA in the disease‐unaffected SOC and none were altered in MSA compared with controls (Fig. 1B).

### 3.2 Phospholipid MUFA are Associated with α‐Synuclein

**FIG 2:**
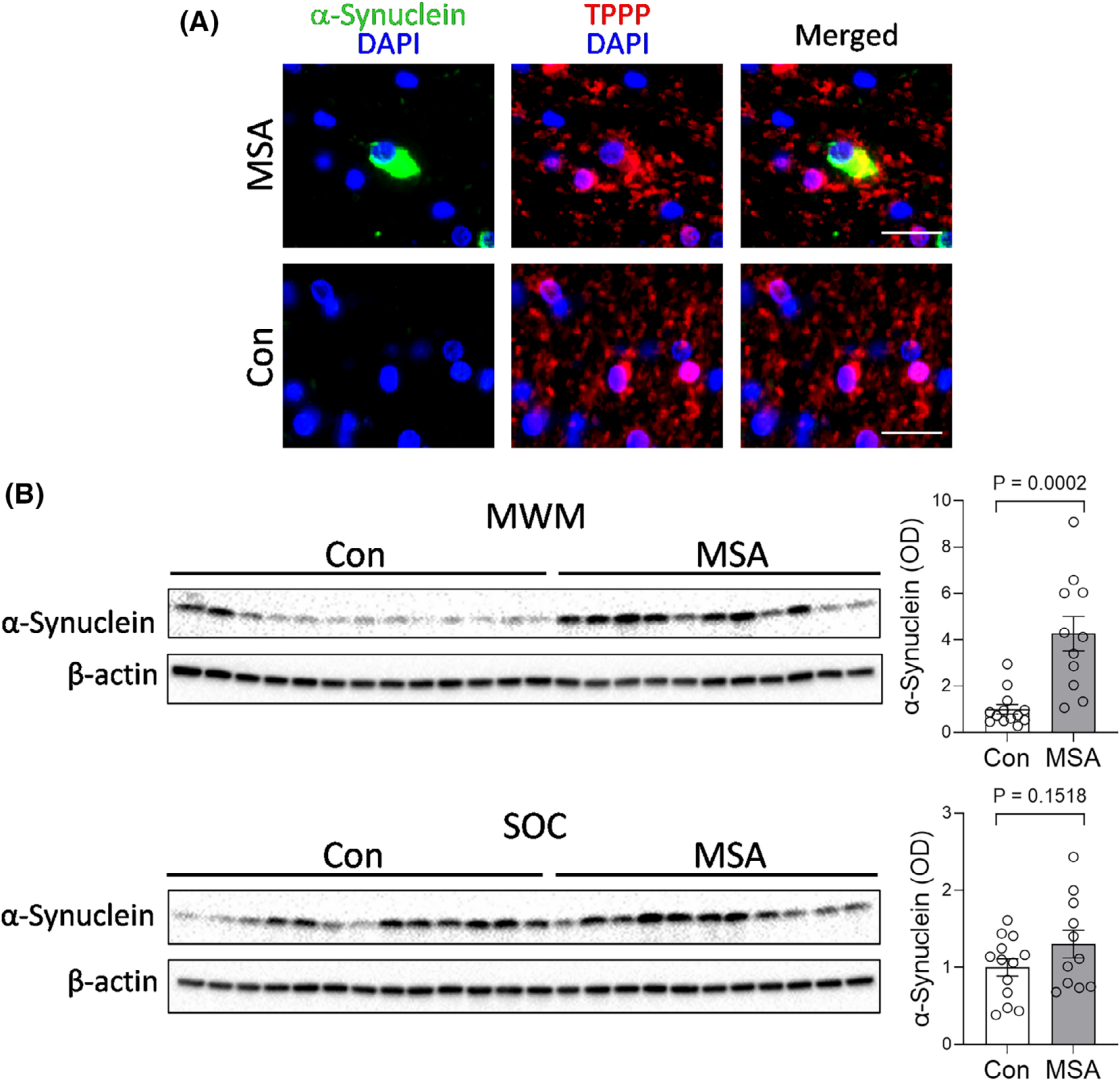
Verification of α‐synuclein inclusion bodies in multiple system atrophy (MSA) brain. (A) Immunofluorescence of α‐synuclein (green), TPPP (red), and cell nucleus (DAPI, blue) in MSA motor cortex white matter (MWM). Scale bar = 20 μm. (B) Western blotting of α‐synuclein in MWM and superior occipital cortex (SOC) normalized with β‐actin and the relative optical density (OD) measurement of α‐synuclein. Data represent mean and standard error of the mean (SEM) as error bars. Con, controls. [Color figure can be viewed at wileyonlinelibrary.com]

**FIG 3:**
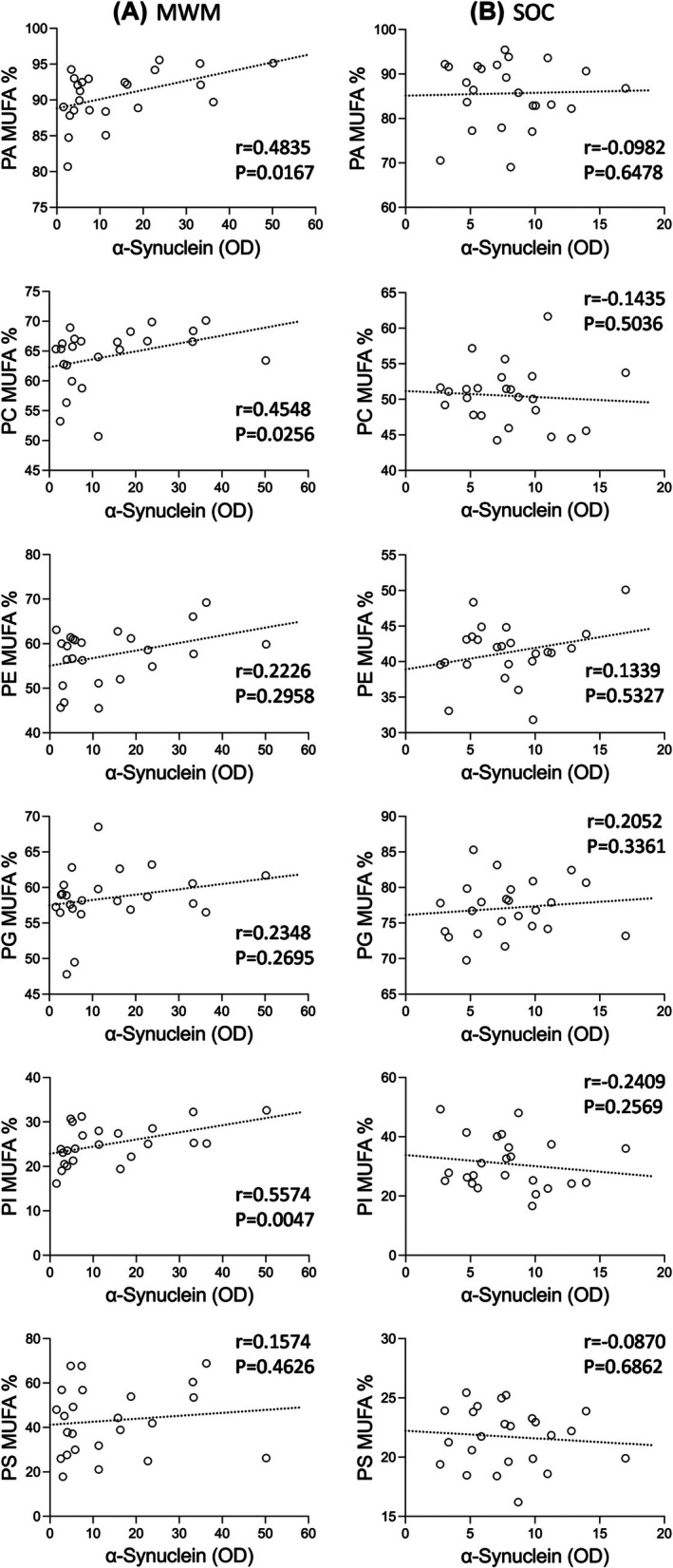
Relationship between phospholipid monounsaturated fatty acids and α‐synuclein. Spearman's correlation of α‐synuclein and percentage of monounsaturated fatty acids (MUFA) within phosphatidic acid (PA), phosphatidylcholine (PC), phosphatidylethanolamine (PE), phosphatidylglycerol (PG), phosphatidylinositol (PI), and phosphatidylserine (PS) in motor white matter (MWM) (A) and superior occipital cortex (SOC) (B). OD, optical density.

We next assessed how the changes to phospholipid MUFA relate to α‐synuclein, the prominent pathogenic protein in MSA. First, we verified the presence of GCI in MSA MWM by immunofluorescence staining (Fig. 2A). We also showed that α‐synuclein levels are significantly increased in MSA MWM compared with controls and unaltered in MSA SOC as measured by Western blotting (Fig. 2B). To understand the relationship between phospholipid MUFA and α‐synuclein we plotted the proportion of MUFA in phospholipids against α‐synuclein levels and found that MUFA in PA, PC, and PI were positively correlated to α‐synuclein levels in MWM, whereas MUFA in PE, PG, and PS showed no trend (Fig. 3A). In contrast, none of the phospholipid MUFA were correlated to α‐synuclein levels in SOC (Fig. 3B). When put together, these results suggest that phospholipid MUFA are elevated in disease‐affected regions of MSA brain that relate to increasing tissue levels of α‐synuclein.

### 3.3 SCD Expression is Downregulated in MSA Brain

**FIG 4:**
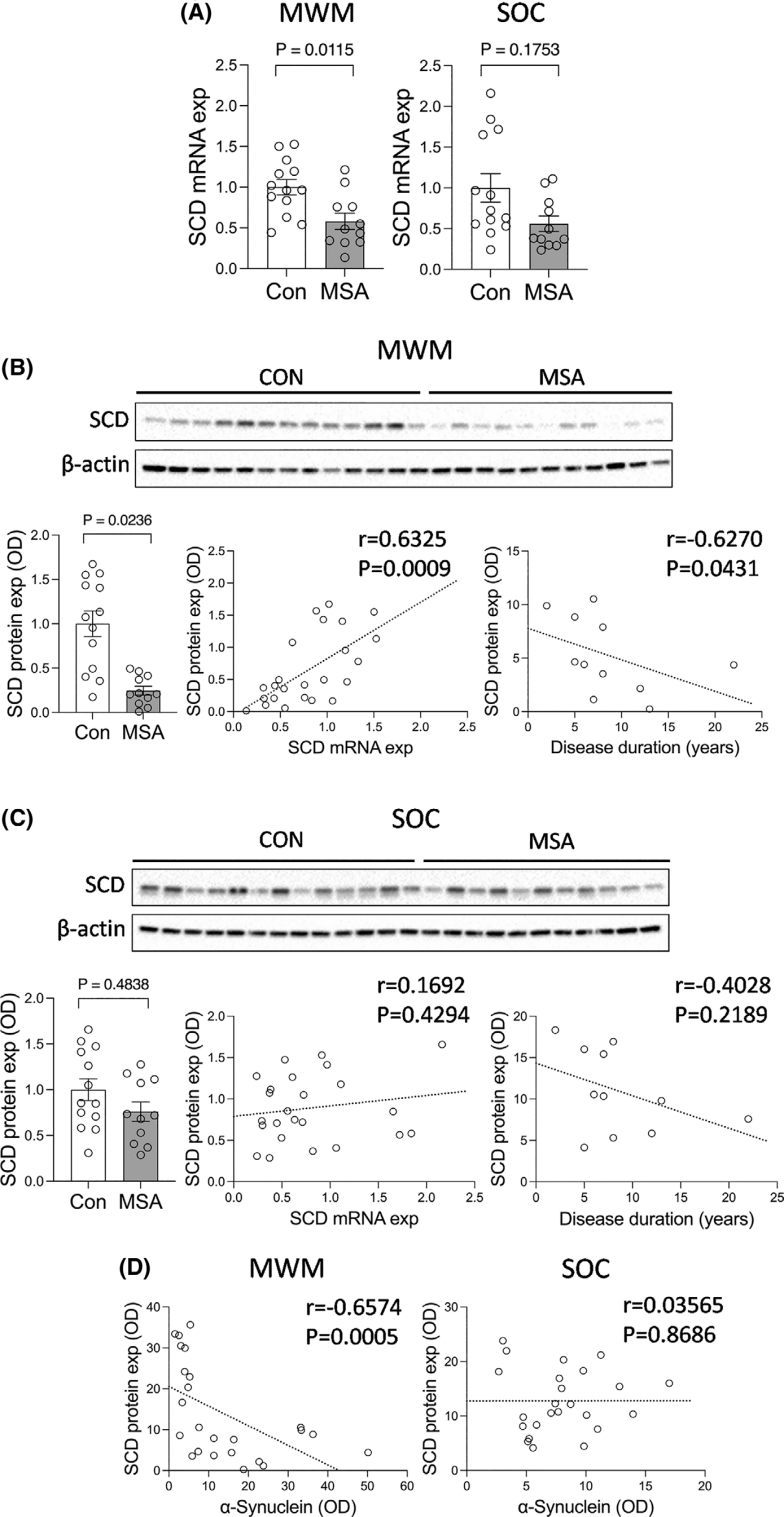
Analysis of stearoyl‐CoA desaturase (SCD) expression in multiple system atrophy (MSA) brain. (A) SCD mRNA expression in motor cortex white matter (MWM) and superior occipital cortex (SOC). (B) SCD protein expression in MWM and optical density (OD) measurement of SCD protein band. Correlation between SCD protein and SCD mRNA in MWM. Correlation between SCD protein and disease duration in MWM. (C) SCD protein expression in SOC and OD measurement of SCD protein band. Correlation between SCD protein and SCD mRNA in SOC. Correlation between SCD protein and disease duration in SOC. (D) Correlation between SCD protein and α‐synuclein protein in MWM and SOC. Data represent mean and standard error of the mean (SEM) as error bars. Con, controls.

Since phospholipid MUFA levels are elevated in MSA brain and the fact that MUFA are synthesized by the enzyme SCD, we were interested in whether SCD expression is altered in MSA brain. We measured the expression of SCD at the mRNA level and found that it was significantly decreased in MSA MWM compared with controls and unchanged in MSA SOC (Fig. 4A). We then measured the expression of SCD at the protein level and found that it was also significantly decreased in MSA MWM compared with controls and unchanged in MSA SOC (Fig. 4B). The SCD mRNA and the SCD protein levels were strongly correlated with each other only in MWM (Fig. 4B,C). The SCD protein levels were inversely associated with MSA disease duration only in MWM (Fig. 4B,C). We also assessed the relationship between SCD protein and α‐synuclein levels and found that SCD protein was inversely associated with α‐synuclein levels only in MWM (Fig. 4D).

### 3.4 Analysis of SCD in MSA Blood

**FIG 5:**
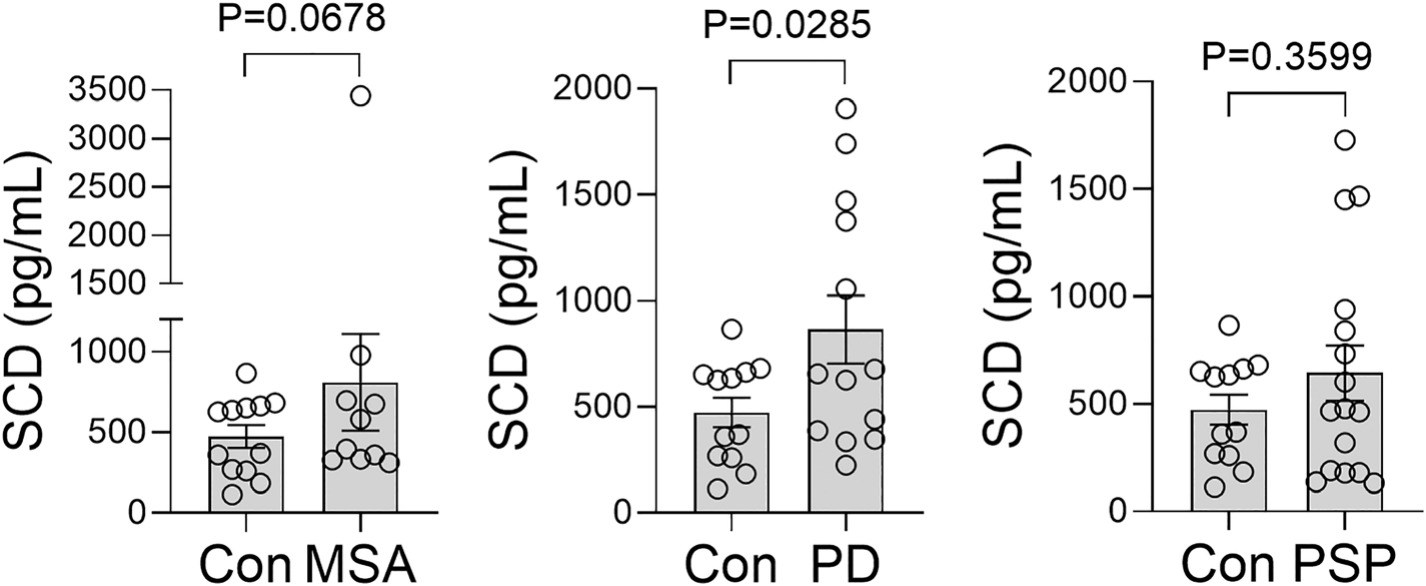
Measurement of stearoyl‐CoA desaturase in multiple system atrophy (MSA) blood serum. Serum stearoyl‐CoA desaturase (SCD) concentrations in MSA, Parkinson's disease (PD), progressive supranuclear palsy (PSP), and controls (Con) were measured by ELISA. Data represent mean and standard error of the mean (SEM) as error bars.

Finally, in addition to understanding the contribution of SCD in MSA pathogenesis, we were interested in whether SCD could be measured in blood serum to explore the idea of using SCD as a peripheral biomarker for MSA. For this study, we analyzed serum collected from a cohort of MSA and two other movement diseases, namely PD (a positive control for synucleinopathy) and PSP (a negative control for synucleinopathy). Serum SCD was measured by ELISA, covarying for age, sex, and MSA‐C/P subtype (in the case of MSA). SCD was measurable in serum, with the SCD concentration detected in the range of 112–3440 pg/mL. However, the difference between MSA and controls did not reach significance, whereas it significantly increased in PD, but not in PSP (Fig. 5).

## 4 Discussion

MSA is an aggressive neurodegenerative disease pathologically characterized by the presence of GCI‐containing α‐synuclein aggregates. Many data indicate that binding of α‐synuclein to membrane lipids is an early event that precedes the formation of α‐synuclein aggregates. Membranes, including myelin, are mainly composed of phospholipids with various fatty acids, including MUFA. Studies of MUFA in the context of PD demonstrate that increases in their abundance induce α‐synuclein aggregation and toxicity.^12^, ^16^ However, very little is known about MUFA in the context of MSA pathology. We therefore undertook a comprehensive assessment of MUFA in phospholipids in different regions of MSA brain. We found that the proportions of MUFA in PA, PC, and PE were elevated in the disease‐affected MWM, whereas they were unaltered in the disease‐unaffected SOC. Importantly, MUFA were strongly associated with increasing levels of α‐synuclein in MWM where disease inclusions are forming. These results suggest that MUFA could be related or contribute to MSA pathology, as is the case with PD.

MUFA are synthesized by the enzyme SCD, and multiple lines of evidence indicate that inhibition of SCD reduces α‐synuclein toxicity in PD models, and thus SCD inhibitors have been trialed in PD patients.^8^, ^9^, ^10^, ^11^ Despite this, no such studies of SCD in MSA nor clinical trials in MSA patients have been reported. We therefore assessed SCD expression in the two regions of MSA brain and found that both mRNA and protein levels were decreased in MSA MWM compared with controls, likely as a feedback inhibition due to the high levels of MUFA in that region. Furthermore, SCD protein levels were inversely associated with disease duration and the increasing levels of α‐synuclein only in MWM. There were clear regional differences in the change of phospholipid MUFA and the subsequent changes in SCD expression. Based on our evidence, it is plausible to think that these differences could have resulted from differences in the α‐synuclein level in different regions of MSA brain. The increased level of α‐synuclein in the disease‐affected MWM would alter the interaction between α‐synuclein and phospholipids and modify membrane structure.^27^ When put together, these results suggest a prevalence of MUFA dysregulation associated with disease pathology in specific regions of MSA brain, resulting in MUFA levels remaining high despite the decreases in SCD expression. It is unclear what is causing MUFA dysregulation in MSA and how it contributes to MSA pathogenesis. In an earlier study,^6^ a number of lipid classes were shown to be altered in the same disease‐affected region (ie, MWM) but not in the disease‐unaffected SOC, and therefore we can only speculate that MUFA dysregulation is a part and parcel of general lipid aberration in disease‐affected regions of MSA brain. It is interesting to note, however, that in MS, which is also a demyelinating disease, decreases in SCD expression^22^ are thought to cause myelin instability and fragmentation.^21^

The pathological effects of MUFA stem from the fact that α‐synuclein has a higher affinity to phospholipids containing MUFA compared with saturated fatty acids.^28^, ^29^ Increases in MUFA content in phospholipids cause physicochemical changes to membranes, such as elevated membrane fluidity, and these are thought to enhance α‐synuclein–lipid interaction and subsequent conformational changes to α‐synuclein monomers that render them predisposed to form aggregates.^30^, ^31^ The strong association observed between MUFA‐rich phospholipids and α‐synuclein levels supports the notion that increased levels of MUFA content in phospholipids increase α‐synuclein toxicity and this may contribute to MSA pathogenesis. We showed that of all the phospholipid MUFA that were increased in MSA MWM, PA MUFA had the strongest association with α‐synuclein levels. These results are consistent with a previous in vitro study that showed that α‐synuclein binds strongly to PA MUFA and promotes α‐synuclein secondary structural changes and aggregation formation.^32^ In one study, the PA 18:1/18:1 species was shown to provide the strongest binding affinity for α‐synuclein, increasing α‐synuclein oligomerization and proteinase K‐resistance.^33^ It is interesting to note that, unlike other phospholipids, PA is only a minor structural component of membranes. It has a secondary role as a messenger, regulating a wide variety of cellular processes, such as signal transduction and cellular differentiation.^34^ Therefore, it is reasonable to think that increases in PA MUFA may affect α‐synuclein pathology, indirectly, by involving other downstream processes.

In the context of MS, which also impacts oligodendroglia and has reduced SCD (as seen in MSA), the application of a SCD inhibitor increased colocalization of myelin basic protein (MBP) and neurofilament and promoted myelination of demyelinated axons in ex vivo mouse brain slices.^35^ Furthermore, deletion of SCD in mice resulted in increases in MBP reactivity and thicker myelin sheaths in vivo,^35^ suggesting that SCD inhibition protects oligodendroglial dysfunction in MS. SCD inhibitors for α‐synuclein PD pathology have been developed^11^, ^15^ and shown to be particularly effective in crossing the blood–brain barrier and reducing fatty acid desaturation index with good pharmacokinetic properties in rats and *Macaca fascicularis* monkeys.^20^ In a PD mouse model, a SCD inhibitor was able to prevent progressive motor deficits and restore dopaminergic integrity and neuronal survival.^10^ While the use of SCD inhibitors to alleviate α‐synuclein toxicity in PD is neuron‐centric, its success in MS suggests SCD inhibitors are also effective for oligodendroglia. SCD is abundant in myelinating oligodendrocytes^15^, ^36^ and is an essential neurotrophic factor^15^, ^21^ with oligodendrocytes containing α‐synuclein aggregates displaying reduced myelin‐related proteins and compromised neurotrophic capacity.^37^ Given our data in MSA, future studies should examine whether SCD inhibition provides similar modulation for oligodendrocytes with α‐synuclein aggregates as seen in MS or in neurons with α‐synuclein aggregates in PD.

There were limitations to our study. First, the MSA and control cohorts could not be aged‐matched, as MSA patients clearly have a shorter lifespan than controls. To address this, we used age as a covariate in all our analysis. Second, the small cohort size of MSA, which stems from the fact that MSA is a rare disease and the donation of MSA brain to brain banks is very low. Future studies of larger cohorts of MSA would be of much benefit.

In summary, we have demonstrated, for the first time, that increased MUFA in phospholipids in disease‐affected regions of MSA brain is associated with increasing α‐synuclein levels and decreasing levels of their synthesizing enzyme SCD. These results implicate MUFA dysregulation and SCD perturbation in increased α‐synuclein toxicity in MSA. Our study has provided new insights into an unrecognized pathway in MSA and opened a new area of research for the better understanding of MSA pathogenesis. Future studies could explore whether modulating MUFA levels alters α‐synuclein pathology in MSA models, and whether the currently reported SCD inhibitors exert beneficial effects on MSA patients.

## Author Roles

(1) Research Project: A. Conception and Development, B. Organization, C. Execution, D. Data Analysis, E. Supervision, F. Provided Expert Advice; (2) Statistical Analysis: A. Design, B. Execution, C. Review and Critique; (3) Manuscript Preparation: A. Writing of the First Draft, B. Revision, C. Review and Critique; (4) Other: A. Lipid Analysis, B. Quantitative Polymerase Chain Reaction (qPCR), C. Western Blotting, D. Immunohistochemistry, E. Liquid Chromatography–Mass Spectrometry (LC–MS) Analysis, F. Provided Serum Samples, G. Provided Laboratory and Salary Funding.

F.I.I.: 1D, 3A, 4A, 4B, 4C.

Y.F.: 3B, 4D.

R.P.: 3B, 4E.

Q.C.: 1F, 3C.

Y.Y.: 1F, 3C.

S.J.G.L.: 1F, 3B, 4F.

N.D.: 1F, 3B, 4F.

G.M.H.: 3B, 4F, 4G.

W.S.K.: 1A, 1D, 1E, 3A.

## Financial Disclosures

This work was supported by funding from Defeat MSA Alliance to W.S.K. F.I.I. was supported by the University of Sydney Research Training Program scholarship. G.M.H. was supported the National Health and Medical Research Council of Australia (Investigator Grant 1176607). Y.F. is an awardee of the University of Sydney BISA grant.

## Data Availability

Data are available on reasonable request upon relevant ethical approval by contacting the corresponding author.
